# Comparison of outcomes after transcatheter aortic valve replacement between elderly (65–79 years) and super-elderly (≥80 years) patients

**DOI:** 10.1097/MD.0000000000029816

**Published:** 2022-06-30

**Authors:** Seok Oh, Ju Han Kim, Cho-Hee Hwang, Dae Young Hyun, Kyung Hoon Cho, Min Chul Kim, Doo Sun Sim, Young Joon Hong, Youngkeun Ahn, Myung Ho Jeong

**Affiliations:** a Department of Cardiology, Chonnam National University Hospital, Gwangju, Korea; b Regional Cardiocerebrovascular Center, Chonnam National University Hospital, Gwangju, Korea; c Department of Cardiology, Chonnam National University Medical School, Gwangju, Korea.

**Keywords:** aged, aortic valve, aortic valve disease, aortic valve stenosis, transcatheter aortic valve replacement

## Abstract

Transcatheter aortic valve replacement (TAVR) is an effective treatment option for patients with severe symptomatic aortic stenosis. Nonetheless, there is a paucity of data regarding the differences in the clinical outcomes of TAVR procedures between elderly and super-elderly patients. This study aimed to compare the clinical characteristics and outcomes of patients aged 65 to 79 years and ≥80 years who underwent TAVR for aortic stenosis.

The clinical characteristics and outcomes of 134 patients with aortic stenosis who underwent TAVR were analyzed. Patients were categorized into 2 groups: an elderly group (EG; 65–79 years) and a super-elderly group (SEG) (≥80 years). The in-hospital and follow-up clinical outcomes were compared between the 2 groups.

The EG tended to be more overweight, obese, and diabetic than the SEG, whereas the SEG had a higher surgical risk but lower creatinine clearance, hematocrit level, and effective orifice area than the EG. However, no difference was found in in-hospital clinical outcomes between the 2 groups, except for atrial fibrillation. In the propensity score matching and inverse probability of treatment weighting-adjusted analyses, these results were similar. All follow-up clinical outcomes were similar, except for rehospitalization, which was statistically attenuated after propensity score matching and inverse probability of treatment weighting-adjusted analyses.

TAVR was associated with similar safety outcomes in the EG (65–79 years) and the SEG (≥80 years). Advanced age is not negatively associated with clinical outcomes after the TAVR procedure.

## 1. Introduction

Aortic stenosis (AS) represents one of the most common valvular heart diseases among older adults, with a prevalence of 5.2% in individuals aged >75 years.^[1]^ AS is a progressive disease characterized by thickening and calcification of the aortic valve (AV), with restricted valve leaflet motion, resulting in left ventricular outflow obstruction.^[1,2]^ In patients with symptomatic AS, the 2-year mortality rate is >50% unless AV replacement is performed.^[3,4]^ Because no medical treatment has been established to prevent or slow its progression, AV replacement is the only primary treatment option for severe symptomatic AS.^[5]^

Following the revolutionary advent of transcatheter AV replacement (TAVR) in 2002,^[6]^ TAVR has been considered a good alternative to surgical AV replacement (SAVR) in patients with severe symptomatic AS. As some randomized controlled trials (RCTs) have demonstrated that TAVR is noninferior (or even superior) to SAVR in patients at high or intermediate surgical risk, these trials have expanded the clinical indications for TAVR to a larger pool of eligible patients.^[7]^ In several RCTs, favorable outcomes have also been reported in patients at low surgical risk. In particular, the PARTNER 3 and Evolut Low Risk trials demonstrated the safety and efficacy of TAVR in eligible patients at low surgical risk.^[7]^ This led to its approval by the United States Food and Drug Administration for the treatment of patients with symptomatic AS, regardless of their surgical risk.^[8]^

The increasing burden of cardiovascular disorders in the aging population is associated with an incremental trend in the prevalence of AS.^[9]^ As this prevalence increases from 2.8% in the population aged 60 to 74 years to >13.1% in the population aged ≥75 years,^[10]^ the demand for TAVR is predicted to easily increase in an aging society.^[11]^ Owing to the innovations in public healthcare practices and medicine, South Korea has experienced a rapid increase in life expectancy.^[12]^ In 2017, 17 years after South Korea became an aging society in 2000, >14% of Korean citizens were aged ≥65 years. This transition suggests that South Korea is one of the fastest aging countries worldwide.^[13]^ Therefore, the number of older patients with AS eligible for TAVR is expected to increase exponentially in South Korea.

Among them, the proportion of super-elderly patients, such as octogenarians and nonagenarians, is also expected to increase. Nonetheless, there is a paucity of data regarding differences in the clinical outcomes of TAVR procedures between elderly and super-elderly patients. This study aimed to compare the clinical characteristics and outcomes of patients aged 65 to 79 years and ≥80 years who underwent TAVR for AS.

## 2. Methods

### 2.1. Study design and participants

**Figure 1.**
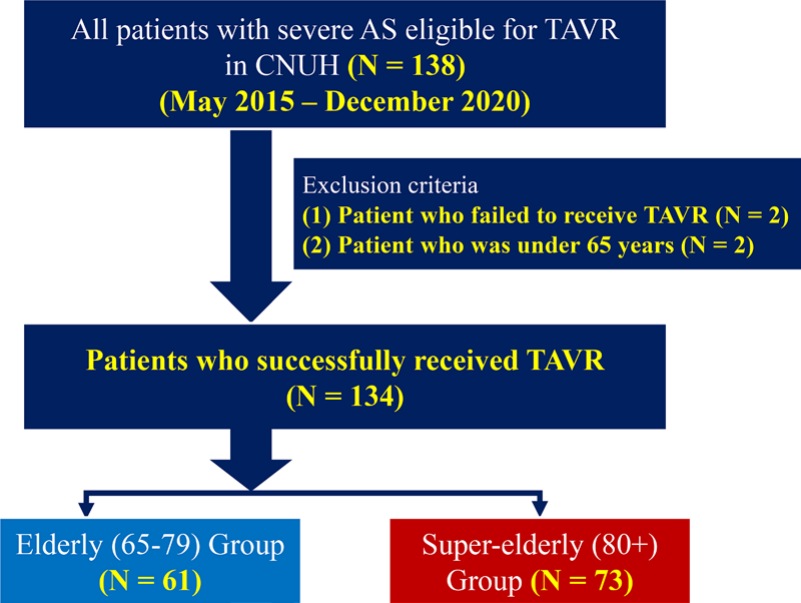
Study flowchart. AS = aortic stenosis, CNUH = Chonnam National University Hospital, TAVR = transcatheter aortic valve replacement.

The study design is shown in Figure 1. Between May 2015 and December 2020, 138 consecutive patients with severe AS who underwent TAVR at our institution were initially selected. AS diagnosis was based on clinical, echocardiographic, and hemodynamic criteria, as directed by contemporary guidelines.^[5,14]^ The exclusion criteria were patients who did not undergo TAVR (n = 2) and patients aged <65 years (n = 2). After excluding these patients, 134 patients were finally enrolled in this study. Patients were categorized into 2 groups: an elderly group (EG; 65–79 years) and a super-elderly group (SEG; ≥80 years). Data were collected in a dedicated case report form by qualified cardiologists and trained registered nurses between May 5, 2015, and December 31, 2020. The data were checked for completeness and quality.

### 2.3. Preprocedural workup and TAVR procedure

Prior to TAVR, an extensive workup was performed to assess the major comorbidities and perform risk stratification. Transthoracic echocardiography (TTE) was used to study hemodynamics and valve anatomy (AS severity, leaflet motion, annular size, and degree of calcification). To evaluate the coronary and peripheral vascular status, all of the participants received computed tomography angiography. Computed tomography angiography reveals anatomical characteristics of the coronary and iliofemoral arteries and provides comprehensive information about the anatomy and geometry of the aortic annulus,^[15]^ which facilitates size selection of the valve prosthesis and determines the feasibility of the transfemoral approach. Although coronary computed tomography angiography may exclude severe coronary artery disease, its efficacy in the assessment of the severity of coronary lesions is limited. Hence, coronary angiography is also routinely performed to confirm the presence and assess the severity of coronary artery disease.^[16]^

For each eligible patient, a multidisciplinary heart team, comprising interventional cardiologists, imaging cardiologists, cardiothoracic surgeons, and anesthesiologists, made decisions regarding the eligibility and feasibility of TAVR. The Society of Thoracic Surgeons Predicted Risk of Mortality (STS-PROM) and European System for Cardiac Operative Risk Evaluation II (EuroSCORE II) scores were used to estimate the perioperative risk of TAVR.^[17,18]^ The transcatheter AV (self-expandable or balloon-expandable valve prosthesis) was selected at the discretion of the operating interventional cardiologist. Most patients underwent transfemoral TAVR. Otherwise, alternative routes, such as trans-subclavian TAVR, were selected. Patients were given 1 of 2 options for TAVR: general anesthesia or local anesthesia with conscious sedation (TAVR minimalist approach). If the valve was deemed to have heavy leaflet/outflow tract calcification, predilation ballooning was considered. In the event of suboptimal AV performance, postdilation ballooning was also performed to improve the conformation of the valve prosthesis to the annulus. In the postoperative period, TTE was routinely performed to assess valve prosthesis function.

### 2.4. Study outcomes

In this study, we analyzed the length of hospital stay, length of hospitalization in the intensive care unit, in-hospital complication rates, and clinical outcomes for each group. In-hospital complications included in-hospital mortality, permanent pacemaker implantation, complete atrioventricular block, pericardial effusion, cardiac tamponade, vascular complications, bleeding complications (Bleeding Academic Research Consortium score ≥2), gastrointestinal bleeding, cerebrovascular accident (CVA), left bundle branch block, atrial fibrillation, ventricular tachycardia or fibrillation, acute kidney injury, renal replacement therapy, cardiopulmonary resuscitation, extracorporeal membrane oxygenation, pneumonia, and urinary tract infection.

After discharge, all patients were scheduled to receive outpatient care with routine serial TTE (at 6, 12, and 24 months). Echocardiographic profiles at each visit and clinical outcomes were comprehensively assessed. Follow-up clinical outcomes included major adverse cardiovascular and cerebral event (MACCE), the composite of death and CVA (death or CVA), death from any cause (cardiac and noncardiac death), CVA, nonfatal myocardial infarction (MI), and rehospitalization. MACCE is defined as the composite of death, CVA, nonfatal MI, and rehospitalization.

### 2.5. Statistical analyses

Data are expressed as the mean ± standard deviation for continuous variables or as numbers and percentages for categorical variables. Continuous variables were analyzed using Student *t* test and analysis of variance test, and categorical variables were analyzed using Pearson chi-square test, Fisher exact test, or linear-by-linear association. A *P* value of <.05 was considered statistically significant.

To examine the differences between the 2 groups, we utilized 2 propensity score-matched models: propensity scoring matching (PSM) and inverse probability of treatment weighting (IPTW). These models had a total of 25 covariates, including male sex, body mass index (BMI), STS-PROM score ≥8, medical history (hypertension, diabetes mellitus, chronic kidney disease, dialysis management, atrial fibrillation, CVA, chronic obstructive pulmonary disease, or asthma; recent MI [<90 days]), smoking history, creatinine clearance <60 mL/min/1.73 m^2^, anemia (hemoglobin <13 g/dL [men] or <12 g/dL [women]), hematocrit <35%, type of transcatheter heart valve, transfemoral approach, type of anesthesia (general vs local anesthesia), predilation, postdilation, valve size, total procedure time ≥90 minutes, type of intraoperative echocardiography, left ventricular ejection fraction <50%, and moderate or severe aortic regurgitation. Patients with missing data for any of these covariates or those with a follow-up interval after hospital discharge of 0 days were excluded from the PSM- and IPTW-adjusted analyses.

Cumulative event analyses were conducted using time-to-event data. Survival curves were estimated using the Kaplan–Meier method and compared using the log-rank test. Patients were censored at the time of the event or last follow-up. Kaplan–Meier curves are depicted for the time of occurrence of the clinical outcomes.

Statistical analyses were conducted using SPSS (version 25.0; SPSS Inc., Armonk, NY).

### 2.6. Ethical statement

All procedures performed in studies involving human participants were in accordance with the ethical standards of the institutional and/or national research committee and with the 1964 Declaration of Helsinki and its later amendments or comparable ethical standards. The study protocol was approved by the Institutional Review Board of our institution of Chonnam National University Hospital (approval number: CNUH-2021-333). The requirement for written informed consent was waived owing to the retrospective nature of the study.

## 3. Results

### 3.1. Baseline characteristics

Among the 134 patients aged ≥65 years who underwent TAVR for severe symptomatic AS between May 2015 and December 2020, 61 were aged 65 to 79 years (EG) and 73 were aged ≥80 years (SEG).^[19]^ Baseline clinical characteristics are shown in Table 1. In the entire cohort, the mean age was 80.78 ± 5.51 years, and 44.03% of patients were male. The mean BMI was 23.58 ± 4.10 kg/m^2^; 34.3% of patients had a BMI ≥25 kg/m^2^. The mean STS-PROM and EuroSCORE II scores were 4.373 ± 2.528 and 3.498 ± 1.841, respectively. The baseline clinical characteristics were similar between the EG and SEG, except for age (76.02 ± 3.25 vs 84.75 ± 3.50 years; *P* < .001), weight (62.28 ± 13.95 vs 54.98 ± 9.07 kg; *P* = .001), BMI (24.78 ± 4.71 vs 22.57 ± 3.21 kg/m^2^; *P* =.002), STS-PROM score (3.388 ± 1.616 vs 5.195 ± 2.850; *P* < .001), EuroSCORE II score (3.067 ± 1.709 vs 3.858 ± 1.882; *P* = .013), and presence of diabetes mellitus (37.7% [n = 23] vs 20.5% [n = 15]; *P* = .028). Baseline laboratory, echocardiographic, and procedural profiles are summarized in Table 2. Regarding laboratory profiles, the SEG had lower creatinine clearance than the EG (55.82 ± 24.32 vs 45.21 ± 17.78 mL/min/1.73 m^2^; *P* = .006) and higher creatinine clearance <60 mL/min/1.73 m^2^ (67.2% [n = 44] vs 80.8% [n = 59]; *P* = .045) than the EG. The proportion of patients with hematocrit <35% was higher in the SEG than in the EG (59.0% [n = 36] vs 76.7% [n = 56]; *P* = .028). Echocardiographic profiles were similar between the 2 groups, except for the effective orifice area (EOA) (0.804 ± 0.173 [in the EG] vs 0.681 ± 0.1.77 cm^2^ [in the SEG]; *P* < .001) and indexed EOA (0.501 ± 0.116 [in the EG] vs 0.442 ± 0.110 cm^2^/m^2^ [in the SEG]; *P* = .003). Regarding procedural profiles, the proportions of patients with general anesthesia (44.3% [n = 27] vs 24.7% [n = 18]; *P* = .017) and intraoperative transesophageal echocardiography (44.3% [n = 27] vs 24.7% [n = 18]; *P* = .017) were higher in the EG than in the SEG, which indicates that the EG underwent less invasive TAVR procedures. For the remaining procedural profiles, both groups were similar. These differences were well balanced after PSM and IPTW adjustments (Tables S1 and S2, Supplemental Digital Content, http://links.lww.com/MD/G835).

**Table 1:** Baseline clinical characteristics.

Characteristics	Overall (n = 134)	Elderly group (n = 61)	Super-elderly group (n = 73)	*P* value
Age (yr)	80.78 ± 5.51	76.02 ± 3.25	84.75 ± 3.50	<.001
Male sex	59 (44.0%)	29 (47.5%)	30 (41.1%)	.454
Weight (kg)	58.30 ± 12.07	62.28 ± 13.95	54.98 ± 9.07	**.001**
Height (cm)	157.01 ± 9.12	158.21 ± 9.51	156.00 ± 8.72	.164
BMI (kg/m^2^)	23.58 ± 4.10	24.78 ± 4.71	22.57 ± 3.21	**.002**
BMI ≥25 kg/m^2^	46 (34.3%)	31 (50.8%)	15 (20.5%)	<.001
Risk score				
STS-PROM score	4.373 ± 2.528	3.388 ± 1.616	5.195 ± 2.850	<.001
STS-PROM ≥8	10 (7.5%)	0 (0.0%)	10 (13.7%)	**.003**
EuroSCORE II	3.498 ± 1.841	3.067 ± 1.709	3.858 ± 1.882	**.013**
Prior medical history				
Hypertension	104 (77.6%)	49 (80.3%)	55 (75.3%)	.491
Diabetes mellitus	38 (28.4%)	23 (37.7%)	15 (20.5%)	**.028**
Chronic kidney disease	13 (9.7%)	8 (13.1%)	5 (6.8%)	.222
on dialysis management	3 (2.2%)	2 (3.3%)	1 (1.4%)	.591
Atrial fibrillation	25 (18.7%)	10 (16.4%)	15 (20.5%)	.539
CVA	25 (18.7%)	10 (16.4%)	15 (20.5%)	.539
COPD or asthma	17 (12.7%)	9 (14.8%)	8 (11.0%)	.511
Recent (<90 d) MI	12 (9.0%)	7 (11.5%)	5 (6.8%)	.350
Smoking history				.492
Current smoker or ex-smoker	21 (15.7%)	11 (18.0%)	10 (13.7%)	
Nonsmoker	113 (84.3%)	50 (82.0%)	63 (86.3%)	

All values are expressed as mean ± standard deviation or the number with percentage (%). The elderly group includes patients aged 65-79 years, and the super-elderly group includes patients aged ≥80 years.

BMI = body mass index, CABG = coronary artery bypass graft, COPD = chronic obstructive pulmonary disease, CVA = cerebrovascular accident, EuroSCORE = The European System for Cardiac Operative Risk Evaluation, MI = myocardial infarction, STS-PROM = The Society of Thoracic Surgeons Predicted Risk of Mortality.

**Table 2:** Baseline laboratory, echocardiographic, and procedural profiles.

	Overall (n = 134)	Elderly group (n = 61)	Super-elderly group (n = 73)	*P* value
Laboratory profiles				
Creatinine (mg/dL)	1.11 ± 1.35	1.30 ± 1.94	0.96 ± 0.39	.179
CrCl (mL/min/1.73 m^2^)	50.04 ± 21.59	55.82 ± 24.32	45.21 ± 17.78	**.006**
CrCl <60 mL/min/1.73 m^2^	101 (75.4%)	41 (67.2%)	60 (82.2%)	**.045**
Hgb (g/dL)	11.25 ± 1.66	11.53 ± 1.68	11.01 ± 1.63	.075
Anemia (Hgb <13 g/dL in men, or <12 g/dL in women)	103 (76.9%)	44 (72.1%)	59 (80.8%)	.235
Hct (%)	32.80 ± 4.69	33.42 ± 5.32	32.27 ± 4.06	.158
Hct <35%	92 (68.7%)	36 (59.0%)	56 (76.7%)	**.028**
Echocardiographic profiles				
LVEF (%)	62.69 ± 11.87	64.00 ± 11.86	61.59 ± 11.84	.243
LVEF <50%	17 (12.7%)	7 (11.5%)	10 (13.7%)	.700
Peak AoV velocity (m/s)	4.74 ± 0.65	4.65 ± 0.57	4.81 ± 0.70	.143
Peak AoV PG (mm Hg)	92.20 ± 25.73	88.04 ± 21.34	95.68 ± 28.59	.087
Mean AoV PG (mm Hg)	53.70 ± 16.92	50.65 ± 13.59	56.24 ± 18.97	.050
EOA (cm^2^)	0.737 ± 0.185	0.804 ± 0.173	0.681 ± 0.177	<.001
Indexed EOA (cm^2^/m^2^)	0.469 ± 0.116	0.501 ± 0.116	0.442 ± 0.110	**.003**
Moderate or severe AR	29 (21.6%)	17 (27.9%)	12 (16.4%)	.110
Procedural profiles				
Type of THV				.681
Self-expandable prosthesis	101 (75.4%)	47 (77.0%)	54 (74.0%)	
Balloon-expandable prosthesis	33 (24.6%)	14 (23.0%)	19 (26.0%)	
Vascular approach				.272
Femoral approach	133 (99.3%)	60 (98.4%)	73 (100.0%)	
Nonfemoral approach	1 (0.7%)	1 (1.6%)	0 (0.0%)	
Type of anesthesia				**.017**
General anesthesia	45 (33.6%)	27 (44.3%)	18 (24.7%)	
Local anesthesia	89 (66.4%)	34 (55.7%)	55 (75.3%)	
Predilation ballooning	27 (20.1%)	9 (14.8%)	18 (24.7%)	.155
Postdilation ballooning	19 (14.2%)	9 (14.8%)	10 (13.7%)	.862
Valve size (mm)	28.10 ± 2.62	28.00 ± 2.43	28.19 ± 2.78	.675
Total procedure time (min)	93.92 ± 30.14	97.05 ± 31.34	91.30 ± 29.05	.273
Total procedure time ≥90 min	81 (60.4%)	38 (62.3%)	43 (58.9%)	.689
Intraoperative echocardiography				**.017**
TTE guidance	45 (33.6%)	27 (44.3%)	18 (24.7%)	
TEE guidance	89 (66.4%)	34 (55.7%)	55 (75.3%)	

All values are expressed as mean ± standard deviation or the number with percentage (%). The elderly group includes patients aged 65-79 years, and the super-elderly group includes patients aged ≥80 years.

AoV = aortic valve, AR = aortic regurgitation, CrCl = creatinine clearance, EOA = effective orifice area, Hct = hematocrit, Hgb = hemoglobin, LVEF = left ventricular ejection fraction, PG = pressure gradient, TEE = transesophageal echocardiography, THV = transcatheter heart valve, TTE = transthoracic echocardiography.

### 3.2. In-hospital clinical outcomes

Significant differences were not found in the duration of hospital stay and in-hospital complication rates between the 2 groups, except for atrial fibrillation. Atrial fibrillation was more common in the SEG than in the EG (15.1% [n = 11] vs 1.6% [n = 1]; *P* = .006; Table 3). Regarding propensity score weighting (PSM- and IPTW-adjusted analyses), the results were similar between the 2 groups, except for a Bleeding Academic Research Consortium score ≥2 in the post-IPTW analysis (Table S3, Supplemental Digital Content, http://links.lww.com/MD/G835).

**Table 3:** Duration of hospital stay and in-hospital complications.

	Overall (n = 134)	Elderly group (n = 61)	Super-elderly group (n = 73)	*P* value
Duration of hospital stay				
Length of hospital stay	15.43 ± 12.62	14.34 ± 11.81	16.33 ± 13.26	.367
Length of ICU hospitalization	2.24 ± 1.73	2.08 ± 1.23	2.37 ± 2.05	.338
In-hospital complications				
In-hospital death	6 (4.5%)	2 (3.3%)	4 (5.5%)	.540
CAVB	19 (14.2%)	9 (14.8%)	10 (13.7%)	.862
PPM implantation	17 (12.7%)	7 (11.5%)	10 (13.7%)	.700
Atrial fibrillation	12 (9.0%)	1 (1.6%)	11 (15.1%)	**.006**
New-onset LBBB	62 (46.3%)	32 (52.5%)	30 (41.1%)	.189
VT or VF	7 (5.2%)	2 (3.3%)	5 (6.8%)	.454
Pericardial effusion	13 (9.7%)	5 (8.2%)	8 (11.0%)	.591
Cardiac tamponade	1 (0.7%)	0 (0.0%)	1 (1.4%)	1.000
BARC ≥2	13 (9.7%)	9 (14.8%)	4 (5.5%)	.085
Bleeding complications				.100
BARC 0	109 (81.3%)	48 (78.7%)	61 (83.6%)	
BARC 1	12 (9.0%)	4 (6.6%)	8 (11.0%)	
BARC 2	7 (5.2%)	4 (6.6%)	3 (4.1%)	
BARC 3	5 (3.7%)	4 (6.6%)	1 (1.4%)	
BARC 4	0 (0.0%)	0 (0.0%)	0 (0.0%)	
BARC 5	1 (0.7%)	1 (1.6%)	0 (0.0%)	
Vascular complications	10 (7.5%)	5 (8.2%)	5 (6.8%)	.768
Gastrointestinal bleeding	3 (2.2%)	1 (1.6%)	2 (2.7%)	1.000
Acute kidney injury	14 (10.4%)	7 (11.5%)	7 (9.6%)	.722
Urgent RRT	3 (2.2%)	1 (1.6%)	2 (2.7%)	1.000
CPR	4 (3.0%)	1 (1.6%)	3 (4.1%)	.625
ECMO	1 (0.7%)	0 (0.0%)	1 (1.4%)	1.000
CVA	5 (3.7%)	3 (4.9%)	2 (2.7%)	.659
Pneumonia	5 (3.7%)	2 (3.3%)	3 (4.1%)	1.000
Urinary tract infection	6 (4.5%)	1 (1.6%)	5 (6.8%)	.220

All values are expressed as mean ± standard deviation or the number with percentage (%). The elderly group includes patients aged 65-79 years, and the super-elderly group includes patients aged ≥80 years.

BARC = Bleeding Academic Research Consortium, CAVB = complete atrioventricular block, CPR = cardiopulmonary resuscitation, CVA = cerebrovascular accident, ECMO = extracorporeal membrane oxygenation, ICU = intensive care unit, LBBB = left bundle branch block, PPM = permanent pacemaker, RRT = renal replacement therapy, VF = ventricular fibrillation, VT = ventricular tachycardia.

### 3.3. Follow-up clinical outcomes

**Figure 2.**
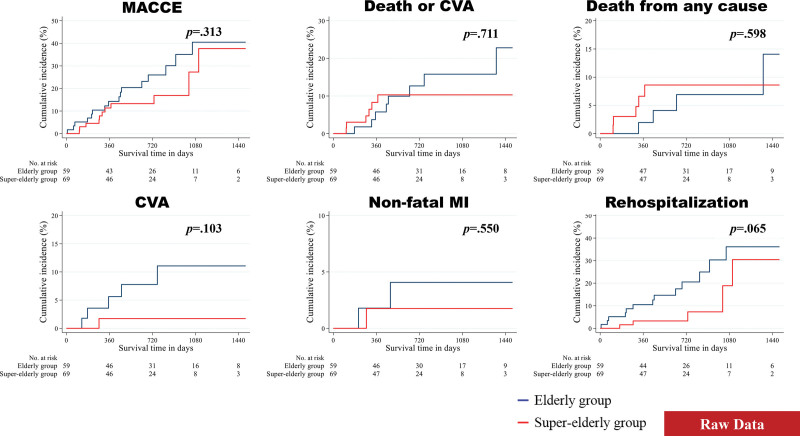
Event rates of follow-up clinical outcomes for all patients (before propensity score matching). The figure shows Kaplan–Meier curves for the cumulative incidence rates stratified by age. CVA = cerebrovascular accident, MACCE = major adverse cardiovascular and cerebral event, MI = myocardial infarction.

**Figure 3.**
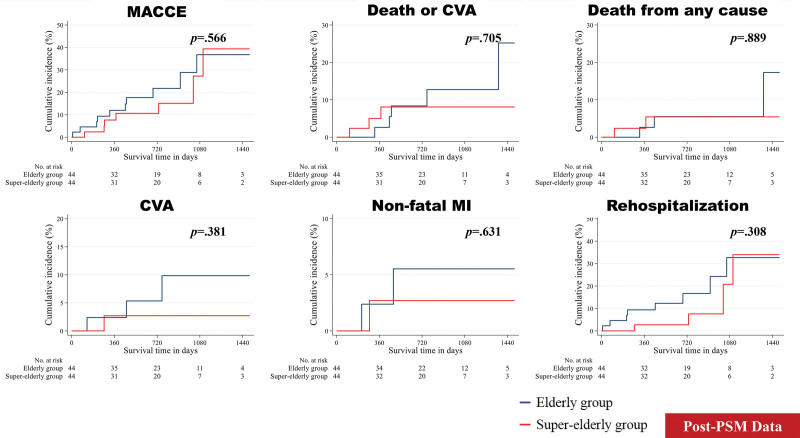
Event rates of follow-up clinical outcomes for all patients (after propensity score matching). The figure shows Kaplan–Meier curves for the cumulative incidence rates stratified by age. CVA = cerebrovascular accident, MACCE = major adverse cardiovascular and cerebral event, MI = myocardial infarction, PSM = propensity score matching.

**Figure 4.**
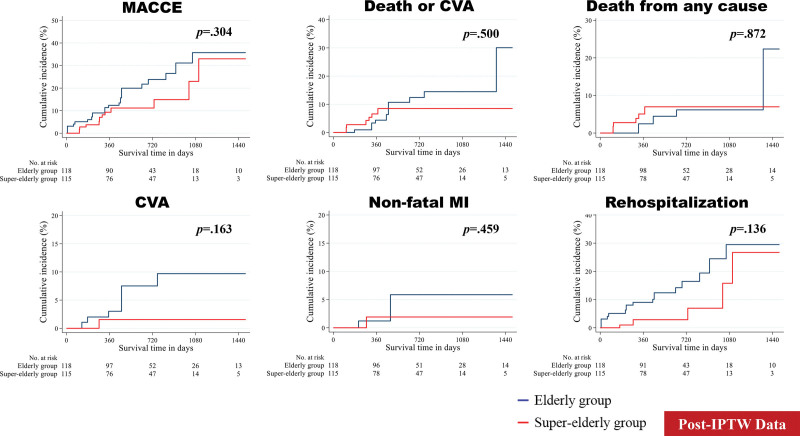
Event rates of follow-up clinical outcomes for all patients (after inverse probability of treatment weighting). The figure shows Kaplan–Meier curves for the cumulative incidence rates stratified by age. CVA = cerebrovascular accident, IPTW = inverse probability of treatment weighting, MACCE = major adverse cardiovascular and cerebral event, MI = myocardial infarction.

After excluding patients who died during the index hospitalization and those with missing data, 128 patients were included in the analysis of follow-up clinical outcomes. Hemodynamic outcomes measured using TTE demonstrated no significant difference between the 2 groups, except for left ventricular ejection fraction at discharge (66.75% ± 9.61% [in the EG] vs 63.10% ± 10.88% [in the SEG]; *P* = .046; Table S4, Supplemental Digital Content, http://links.lww.com/MD/G835). The median follow-up interval for all post-TAVR survivors was 641 (mean, 724.66) days. The clinical outcomes, including MACCE, the composite of death and CVA, death from any cause, CVA, nonfatal MI, and rehospitalization, were determined. The unadjusted, PSM-adjusted, and IPTW-adjusted survival curves were estimated using the Kaplan–Meier method (Figs. 2–4). In the unadjusted analysis, no differences were found, except for rehospitalization. The incidence of rehospitalization was slightly higher in the EG than in the SEG (*P* = .065). In the PSM- and IPTW-adjusted analyses, all follow-up clinical outcomes were similar between the 2 groups.

## 4. Discussion

**Figure 5.**
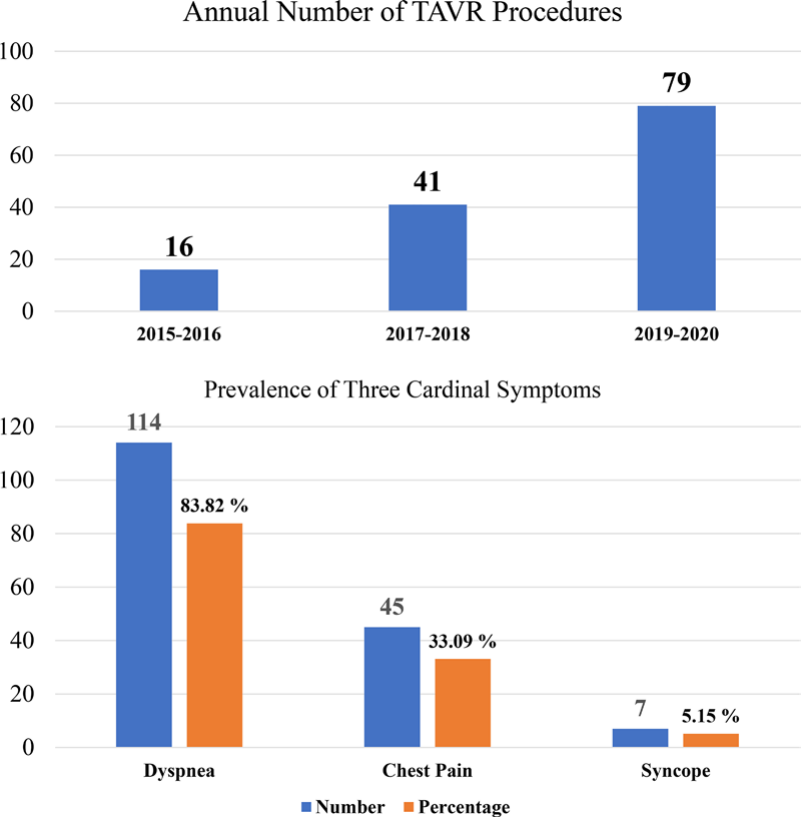
Information on number of procedures and cardinal symptoms of study population. (A) Annual number of TAVR procedures in CNUH. (B) Prevalence of 3 cardinal symptoms among TAVR-treated AS patients in CNUH. AS = aortic stenosis, CNUH = Chonnam National University Hospital, TAVR = transcatheter aortic valve replacement.

The first TAVR procedure in Chonnam National University Hospital, a high-volume national tertiary medical institution located in the southwestern part of South Korea, began on May 5, 2015. Since then, the number of TAVR procedures has gradually increased, from 16 in 2015–2016 to 77 in 2019–2020 (Fig. 5A). In these 136 patients, dyspnea was the most common symptom (83.82%), followed by chest pain (33.09%) and syncope (5.15%), which is similar to the results of a study conducted in Japan (Fig. 5B). Given that the high proportion of dyspnea, known to be associated with the worst prognosis, was noted and symptomatic patients require urgent intervention (TAVR or SAVR) in severe AS, TAVR can be considered one of the appropriate treatments.

Our study compared patients aged 65 to 79 years (EG) with those aged ≥80 years (SEG) who underwent TAVR at our institution between May 2015 and December 2020. The EG patients tended to be more overweight, obese, and diabetic than the SEG patients; however, they had a lower surgical risk. The SEG patients had poorer kidney function than the EG patients. Although hemoglobin levels were similar between the 2 groups, the SEG patients were more anemic and had a lower hematocrit level than the EG patients. The SEG patients had more advanced features of AS, with lower EOA and indexed EOA values. Considering that local anesthesia and TTE guidance were more frequently applied in the SEG patients, they can be inferred to have received less invasive TAVR procedures than the EG patients. Despite these differences, in-hospital complication rates were similar in both groups, except for atrial fibrillation. After propensity score weighting, all in-hospital complication rates were similar in both groups. When referencing a nationwide observational study conducted in Germany,^[20]^ most in-hospital treatment estimates tend to have similar incidences, although the incidence of in-hospital death (2.6%–2.9% vs 4.5%) was slightly higher. Although the incidence of rehospitalization was nonsignificantly higher in the EG, all follow-up clinical outcomes were similar in both groups, with or without propensity score weighting.

Degenerative AS represents the most frequent type of valvular heart disease worldwide. SAVR has traditionally been the recommended treatment for symptomatic AS, alleviating symptoms and prolonging life expectancy. TAVR, a novel alternative, has expanded its clinical indications, whereas a series of large-scale landmark RCTs demonstrated good performance in terms of safety and efficacy.^[7]^ With major evolutionary changes in patient selection, procedural techniques, and device technology, TAVR has shown clinical outcomes similar (or somewhat superior) to SAVR. According to a review by the Society of Thoracic Surgeons–American College of Cardiology Transcatheter Valve Therapy Registry, the annual volume of TAVR has increased^[21]^ and has surpassed that of isolated SAVR in 2016 and all forms of SAVR after the United States Food and Drug Administration’s recent approval of TAVR for patients at low surgical risk. The number of medical institutions performing TAVR and the mean annual volume per site have also steadily increased. While the annual number of patients at high or extremely high surgical risk who undergo TAVR has remained high, the number of patients at low or intermediate surgical risk has gradually increased. In the United States, a total of 8395 patients at low surgical risk underwent TAVR in 2019, accounting for 11.5% of all TAVR cases. The TAVR procedure rate is expected to increase exponentially, and the annual volume worldwide is predicted to reach approximately 300,000 cases by 2025.^[22]^

In addition to its rapidly increasing use worldwide, TAVR is becoming a well-known treatment alternative for patients with severe symptomatic AS. Considering that South Korea, an aged society, is expected to progress to a super-aged society in the future,^[23]^ the number of super-elderly patients aged ≥80 years who are eligible for TAVR is likely to increase dramatically. As current guidelines recommend TAVR for patients with severe symptomatic AS aged ≥80 years over SAVR, the demand for TAVR in aging populations is expected to rapidly increase. In this study, TAVR showed similar outcomes between patients aged 65 to 79 years and ≥80 years, even though super-elderly patients were at higher surgical risk than elderly patients. This finding indicates that TAVR is a relatively safe procedure, even in patients aged ≥80 years.

The present study demonstrates similar outcomes in patients treated with TAVR, regardless of age. In some RCTs, age was not found to be an independent determinant of all-cause mortality. In the PARTNER trial cohort B, which demonstrated better outcomes in TAVR than in medical treatment among severe AS patients with contraindications for SAVR, approximately 46% of enrolled patients were aged >85 years and they showed comparable benefits from TAVR than patients aged ≤85 years.^[24]^ Some RCTs comparing TAVR and SAVR in patients at high surgical risk included 47% of patients aged >85 years and showed that both age groups benefited from TAVR to a similar extent.^[25,26]^ Some single- and multicenter observational registries also demonstrated that age did not significantly affect the clinical outcomes of patients receiving TAVR.^[27,28]^ Previous studies have shown similar trends to those of our findings. Van der Kley et al^[29]^ reported a study demonstrating similar short- and mid-term clinical outcomes in patients aged ≤80 and >80 years. Furthermore, another comparative study evaluating 5-year clinical outcomes after the TAVR procedure between octogenarian patients and younger patients also showed comparable long-term survival rates.^[30]^ Considering the results of previous clinical studies and the present study, TAVR can be considered a safe and feasible procedure in octogenarian or nonagenarian patients, as in patients of younger age.

Our study had several limitations. First, it was based on the data from a single tertiary center in South Korea. Although our institution is considered a high-volume tertiary referral hospital (>1000 hospital beds with a 24-hour accident and emergency service),^[31]^ it has had less experience in TAVR procedures (<150 cases as of 2020), which means that this study is likely to have low statistical power with small sample size. Therefore, generalizing our clinical outcomes, including in-hospital complication rates and follow-up clinical outcomes, with respect to all TAVR-capable medical centers, is difficult. Second, no data were available on prescribed medications. Analyses of medical treatments that could affect clinical outcomes were not included in this study. Third, the frailty score was also not included in the analysis, even though the importance of frailty status in TAVR survival has been emphasized in the literature. Green et al^[32]^ reported that frailty status, consisting of grip strength, gait speed, activities of daily living, and serum albumin level, was associated with survival rate after TAVR procedures. In general, the SEG is expected to have a worse frailty status than the EG. Although this trend may have resulted in some differences in mortality between the 2 groups, no differences in follow-up clinical outcomes were observed in this study. At last, this was a nonrandomized study. Although we attempted to reduce selection bias using 2 propensity score–weighting models (PSM and IPTW), the number of propensity score-matched patients was not sufficiently large to evaluate the differences between the 2 groups. For these reasons, caution must be exercised when interpreting our clinical results, and a prospective multicenter RCT is needed in the future.

In conclusion, TAVR has similar safety outcomes in patients aged 65 to 79 years and ≥80 years. With the explosive increase in the number of super-elderly patients aged ≥80 years who are eligible for TAVR, it is expected that TAVR will become a well-established and safe alternative to SAVR in the elderly population in the future.

## Acknowledgments

We sincerely thank Cho-Hee Hwang, MPH, a biostatistician, for participating in the statistical analyses of this study. This study was supported by a grant (BCRI21074) from the Chonnam National University Hospital Biomedical Research Institute.

## Author contributions

Conceptualization: Seok Oh.

Data curation: Seok Oh, Kyung Hoon Cho, Min Chul Kim, Doo Sun Sim, Young Joon Hong.

Formal analysis: Seok Oh.

Investigation: Seok Oh, Ju Han Kim.

Software: Seok Oh.

Writing – original draft: Seok Oh.

Writing – review & editing: Kyung Hoon Cho, Min Chul Kim, Doo Sun Sim, Young Joon Hong, Ju Han Kim, Youngkeun Ahn, Myung Ho Jeong.

## Supplementary Material



## References

[1] Nkomo VT Gardin JM Skelton TN. Burden of valvular heart diseases: a population-based study. Lancet. 2006;368:1005–11. 16980116 10.1016/S0140-6736(06)69208-8

[2] Otto CM Prendergast B. Aortic-valve stenosis—from patients at risk to severe valve obstruction. N Engl J Med. 2014;371:744–56. 25140960 10.1056/NEJMra1313875

[3] Kodali SK Williams MR Smith CR. Two-year outcomes after transcatheter or surgical aortic-valve replacement. N Engl J Med. 2012;366:1686–95. 22443479 10.1056/NEJMoa1200384

[4] Makkar RR Fontana GP Jilaihawi H. Transcatheter aortic-valve replacement for inoperable severe aortic stenosis. N Engl J Med. 2012;366:1696–704. 22443478 10.1056/NEJMoa1202277

[5] Otto CM Nishimura RA Bonow RO. 2020 ACC/AHA guideline for the management of patients with valvular heart disease: a report of the American College of Cardiology/American Heart Association joint Committee on Clinical Practice guidelines. Circulation. 2021;143:e72–e227. 33332150 10.1161/CIR.0000000000000923

[6] Cribier A. The development of transcatheter aortic valve replacement (TAVR). Glob Cardiol Sci Pract. 2016;2016:e201632. 28979902 10.21542/gcsp.2016.32 PMC5624190

[7] Bocchino PP Angelini F Alushi B. Transcatheter aortic valve replacement in young low-risk patients with severe aortic stenosis: a review. Front Cardiovasc Med. 2020;7:608158. 33381528 10.3389/fcvm.2020.608158 PMC7767870

[8] Coylewright M Forrest JK McCabe JM. TAVR in low-risk patients: FDA approval, the new NCD, and shared decision-making. J Am Coll Cardiol. 2020;75:1208–11. 32164894 10.1016/j.jacc.2019.12.057

[9] Bowry AD Lewey J Dugani SB. The burden of cardiovascular disease in low- and middle-income countries: epidemiology and management. Can J Cardiol. 2015;31:1151–9. 26321437 10.1016/j.cjca.2015.06.028

[10] De Sciscio P Brubert J De Sciscio M. Quantifying the shift toward transcatheter aortic valve replacement in low-risk patients: a meta-analysis. Circ Cardiovasc Qual Outcomes. 2017;10:e003287. 28600455 10.1161/CIRCOUTCOMES.116.003287

[11] Bergmann T SenGupta PP Narula J. Is TAVR ready for the global aging population? Glob Heart. 2017;12:291–9. 28433492 10.1016/j.gheart.2017.02.002

[12] Yang S Khang YH Harper S. Understanding the rapid increase in life expectancy in South Korea. Am J Public Health. 2010;100:896–903. 20299661 10.2105/AJPH.2009.160341 PMC2853609

[13] Jang IY Lee HY Lee E; 50th Anniversary Committee of Korean Geriatrics Society. Geriatrics fact sheet in Korea 2018 from National Statistics. Ann Geriatr Med Res. 2019;23:50–3. 32743288 10.4235/agmr.19.0013 PMC7387592

[14] Nishimura RA Otto CM Bonow RO. 2014 AHA/ACC guideline for the management of patients with valvular heart disease: a report of the American College of Cardiology/American Heart Association Task Force on practice guidelines. Circulation. 2014;129:e521–643. 24589853 10.1161/CIR.0000000000000031

[15] Blanke P Schoepf UJ Leipsic JA. CT in transcatheter aortic valve replacement. Radiology. 2013;269:650–69. 24261496 10.1148/radiol.13120696

[16] Perry TE George SA Lee B. A guide for pre-procedural imaging for transcatheter aortic valve replacement patients. Perioper Med (Lond). 2020;9:36. 33292498 10.1186/s13741-020-00165-1 PMC7690031

[17] Nashef SA Roques F Michel P. European system for cardiac operative risk evaluation (EuroSCORE). Eur J Cardiothorac Surg. 1999;16:9–13. 10456395 10.1016/s1010-7940(99)00134-7

[18] Puskas JD Kilgo PD Thourani VH. The society of thoracic surgeons 30-day predicted risk of mortality score also predicts long-term survival. Ann Thorac Surg. 2012;93:26–33. 22000786 10.1016/j.athoracsur.2011.07.086

[19] Mouton C Leroux L Bonnet G. Successful management of transcatheter aortic valve implantation by platelet transfusions in a nonagenarian patient with severe autoimmune factor V deficiency. Ann Hematol. 2019;98:1991–2. 30830247 10.1007/s00277-019-03646-6

[20] Gaede L Blumenstein J Liebetrau C. Outcome after transvascular transcatheter aortic valve implantation in 2016. Eur Heart J. 2018;39:667–75. 29228149 10.1093/eurheartj/ehx688 PMC5837346

[21] Carroll JD Mack MJ Vemulapalli S. STS-ACC TVT registry of transcatheter aortic valve replacement. J Am Coll Cardiol. 2020;76:2492–516. 33213729 10.1016/j.jacc.2020.09.595

[22] Cesna S De Backer O Søndergaard L. Rapid adoption of transcatheter aortic valve replacement in intermediate- and high-risk patients to treat severe aortic valve stenosis. J Thorac Dis. 2017;9:1432–6. 28740650 10.21037/jtd.2017.05.67 PMC5506149

[23] Kontis V Bennett JE Mathers CD. Future life expectancy in 35 industrialised countries: projections with a Bayesian model ensemble. Lancet. 2017;389:1323–35. 28236464 10.1016/S0140-6736(16)32381-9 PMC5387671

[24] Leon MB Smith CR Mack M. Transcatheter aortic-valve implantation for aortic stenosis in patients who cannot undergo surgery. N Engl J Med. 2010;363:1597–607. 20961243 10.1056/NEJMoa1008232

[25] Smith CR Leon MB Mack MJ. Transcatheter versus surgical aortic-valve replacement in high-risk patients. N Engl J Med. 2011;364:2187–98. 21639811 10.1056/NEJMoa1103510

[26] Adams DH Popma JJ Reardon MJ. Transcatheter aortic-valve replacement with a self-expanding prosthesis. N Engl J Med. 2014;370:1790–8. 24678937 10.1056/NEJMoa1400590

[27] Mack MJ Brennan JM Brindis R. Outcomes following transcatheter aortic valve replacement in the United States. JAMA. 2013;310:2069–77. 24240934 10.1001/jama.2013.282043

[28] Gilard M Eltchaninoff H Iung B. Registry of transcatheter aortic-valve implantation in high-risk patients. N Engl J Med. 2012;366:1705–15. 22551129 10.1056/NEJMoa1114705

[29] van der Kley F van Rosendael PJ Katsanos S. Impact of age on transcatheter aortic valve implantation outcomes: a comparison of patients aged ≤ 80 years versus patients > 80 years. J Geriatr Cardiol. 2016;13:31–6. 26918010 10.11909/j.issn.1671-5411.2016.01.004 PMC4753009

[30] Kahraman Ay N. Impact of age on long term survival following transcatheter aortic valve implantation. J Geriatr Cardiol. 2019;16:265–71. 31080469 10.11909/j.issn.1671-5411.2019.03.003 PMC6500565

[31] Lee U Kim SE Lee SY. Source analysis and effective control of a COVID-19 outbreak in a University Teaching Hospital during a period of increasing community prevalence of COVID-19. J Korean Med Sci. 2021;36:e179. 34155840 10.3346/jkms.2021.36.e179 PMC8216991

[32] Green P Woglom AE Genereux P. The impact of frailty status on survival after transcatheter aortic valve replacement in older adults with severe aortic stenosis: a single-center experience. JACC Cardiovasc Interv. 2012;5:974–81. 22995885 10.1016/j.jcin.2012.06.011 PMC3717525

