# Supplementary material for: Comparison of outcomes after transcatheter aortic valve replacement between elderly (65–79 years) and super-elderly (≥80 years) patients
Source: Medicine (Baltimore). 2022 Jun 30;101(26):e29816. doi: 10.1097/MD.0000000000029816 (PMC9239624; doi:10.1097/MD.0000000000029816)
Supplement: Supplementary file 1 [file medi-101-e29816-s001.pdf]

Supplemental Table 1. Baseline clinical characteristics after propensity score weighting method.

	After propensity score matching			After inverse probability of treatment weighting		
	**Elderly group (n = 48)**	**Super-elderly group (n = 48)**	**p-value**	**Elderly group (n = 123)**	**Super-elderly group (n = 111)**	**p-value**
**Age (years)**	76.50 ± 2.40	83.69 ± 2.89	**<0.001**	76.78 ± 2.78	83.94 ± 3.01	**<0.001**
**Male gender**	24 (50.0)	21 (43.8)	0.539	49 (40.2)	42 (37.9)	0.826
**Weight (kg)**	60.47 ± 14.61	57.70 ± 8.33	0.258	58.12 ± 12.73	57.53 ± 8.15	0.757
**Height (cm)**	158.45 ± 10.21	157.01 ± 9.04	0.466	156.55 ± 9.58	156.66 ± 8.69	0.954
**BMI (kg/m^2)**	23.95 ± 4.77	23.44 ± 3.07	0.528	23.66 ± 4.36	23.48 ± 3.18	0.827
**BMI ≥ 25 kg/m^2**	19 (39.6)	13 (27.1)	0.194	52 (42.5)	35 (31.3)	0.327
**Risk score**						
**STS-PROM score**	3.41 ± 1.66	3.88 ± 1.33	0.124	4.01 ± 1.91	4.18 ± 1.66	0.739
**STS-PROM ≥ 8**	0 (0.0)	0 (0.0)	-	0 (0.0)	0 (0.0)	-
**EuroSCORE**	3.21 ± 1.83	3.50 ± 1.81	0.436	3.38 ± 2.16	3.83 ± 1.95	0.458
**Prior medical history**						
**Hypertension**	38 (79.2)	38 (79.2)	1.000	100 (81.0)	84 (75.7)	0.539
**Diabetes mellitus**	14 (29.2)	10 (20.8)	0.346	38 (31.0)	31 (27.6)	0.752
**Chronic kidney disease**	5 (10.4)	4 (8.3)	0.726	11 (9.0)	12 (10.6)	0.821
**on dialysis management**	1 (2.1)	0 (0.0)	1.000	2 (2.0)	0 (0.0)	0.201
**Atrial fibrillation**	8 (16.7)	7 (14.6)	0.779	20 (16.6)	14 (12.4)	0.547
**CVA**	9 (18.8)	8 (16.7)	0.789	23 (18.6)	15 (13.7)	0.568
**COPD or asthma**	5 (10.4)	5 (10.4)	1.000	13 (10.3)	10 (9.3)	0.841
**Recent (<90 days) MI**	4 (8.3)	4 (8.3)	1.000	13 (10.3)	9 (7.9)	0.697
**Smoking history**			0.779			0.977

**Current smoker or ex-smoker**	8 (16.7)	7 (14.6)	18 (15.0)	16 (14.8)
**Non-smoker**	40 (83.3)	41 (85.4)	105 (85.0)	95 (85.2)

All values are expressed as mean ± standard deviation or the number with percentage (%).

BMI = body mass index; CABG = coronary artery bypass graft; COPD = chronic obstructive pulmonary disease; CVA = cerebrovascular accident; EuroSCORE = The European System for Cardiac Operative Risk Evaluation; MI = myocardial infarction; STS-PROM = The Society of Thoracic Surgeons Predicted Risk of Mortality.

Supplemental Table 2. Baseline laboratory, echocardiographic, and procedural profiles after propensity score weighting method

	After propensity score matching			After inverse probability of treatment weighting		
	**Elderly group (n = 48)**	**Super-elderly group (n = 48)**	**p-value**	**Elderly group (n = 123)**	**Super-elderly group (n = 111)**	**p-value**
**Laboratory profiles**						
**Creatinine (mg/dL)**	1.10 ± 1.09	0.92 ± 0.35	0.273	1.14 ± 1.48	0.99 ± 0.41	0.370
**CrCl (mL/min/1.73m^2)**	56.19 ± 24.68	50.06 ± 18.88	0.175	53.75 ± 24.98	46.77 ± 18.47	0.158
**CrCl < 60 mL/min/1.73m^2**	33 (68.7)	36 (75.0)	0.496	92 (74.6)	89 (79.6)	0.555
**Hgb (g/dL)**	11.57 ± 1.64	11.44 ± 1.63	0.705	11.27 ± 1.63	11.19 ± 1.67	0.830
**Anemia (Hgb < 13 g/dL in men, or <12 g/dL in women)**	35 (72.9)	35 (72.9)	1.000	94 (76.0)	86 (77.5)	0.865
**Hct (%)**	33.48 ± 5.13	33.26 ± 4.14	0.815	31.95 ± 6.64	32.63 ± 4.06	0.681
**Hct < 35 %**	28 (58.3)	33 (68.7)	0.289	86 (69.7)	80 (72.3)	0.781
**Echocardiographic profiles**						
**LVEF (%)**	63.70 ± 12.53	63.29 ± 10.61	0.864	65.34 ± 11.66	61.49 ± 11.86	0.130
**LVEF < 50 %**	6 (12.5)	4 (8.3)	0.504	11 (8.8)	12 (11.2)	0.667
**Peak AoV velocity (m/s)**	4.59 ± 0.60	4.83 ± 0.64	0.065	4.64 ± 0.54	4.80 ± 0.61	0.150
**Peak AoV PG (mmHg)**	86.82 ± 22.12	96.17 ± 25.25	0.057	87.14 ± 20.12	95.25 ± 24.22	0.063
**Mean AoV PG (mmHg)**	49.74 ± 14.15	55.42 ± 17.29	0.082	50.76 ± 13.33	56.22 ± 17.15	0.090
**EOA (cm^2)**	0.82 ± 0.17	0.71 ± 0.18	**0.006**	0.79 ± 0.17	0.70 ± 0.17	**0.008**
**Indexed EOA (cm^2/m^2)**	0.51 ± 0.12	0.45 ± 0.11	**0.009**	0.51 ± 0.12	0.44 ± 0.10	**0.003**
**Moderate or severe AR**	13 (27.1)	10 (20.8)	0.473	27 (22.2)	22 (19.4)	0.738
**Procedural profiles**						

**Type of THV**			0.816			0.903
**Self-expandable prosthesis**	35 (72.9)	36 (75.0)		87 (71.0)	77 (69.7)	
**Balloon expandable prosthesis**	13 (27.1)	12 (25.0)		36 (29.0)	34 (30.3)	
**Vascular approach**			1.000			0.342
**Femoral approach**	47 (97.9)	48 (100.0)		120 (97.7)	111 (100.0)	
**Non-femoral approach**	1 (2.1)	0 (0.0)		3 (2.3)	0 (0.0)	
**Type of anesthesia**			0.513			0.575
**General anesthesia**	17 (35.4)	14 (29.2)		42 (34.4)	32 (28.8)	
**Local anesthesia**	31 (64.6)	34 (70.8)		81 (65.6)	79 (71.2)	
**Pre-dilation ballooning**	7 (14.6)	8 (16.7)	0.779	17 (13.5)	21 (19.2)	0.437
**Post-dilation ballooning**	8 (16.7)	7 (14.6)	0.779	16 (13.1)	19 (17.2)	0.616
**Valve size (mm)**	28.10 ± 2.55	28.27 ± 2.73	0.758	27.72 ± 2.61	27.80 ± 2.86	0.902
**Total procedure time (minutes)**	94.90 ± 27.65	93.40 ± 28.99	0.796	94.36 ± 31.29	93.89 ± 29.99	0.943
**Total procedure time ≥ 90 minutes**	30 (62.5)	30 (62.5)	1.000	77 (62.2)	71 (64.0)	0.866
**Intraoperative echocardiography**			0.513			0.575
**TTE guidance**	17 (35.4)	14 (29.2)		42 (34.4)	32 (28.8)	
**TEE guidance**	31 (64.6)	34 (70.8)		81 (65.6)	79 (71.2)	

Supplemental Table 3. Duration of hospital stay and in-hospital complications after propensity score weighting method.

	After propensity score matching			After inverse probability of treatment weighting		
	**Elderly group (n = 48)**	**Super-elderly group (n = 48)**	**p-value**	**Elderly group (n = 123)**	**Super-elderly group (n = 111)**	**p-value**
**Duration of hospital stay**						
**Length of hospital stay**	15.38 ± 13.09	15.50 ± 14.49	0.965	15.76 ± 16.52	16.81 ± 13.72	0.778
**Length of ICU hospitalization**	2.10 ± 1.32	2.56 ± 2.37	0.246	2.13 ± 1.20	2.68 ± 2.40	0.242
**In-hospital complications**						
**In-hospital death**	2 (4.2)	2 (4.2)	1.000	7 (5.5)	5 (5.0)	0.921
**CAVB**	9 (18.7)	6 (12.5)	0.399	18 (15.1)	19 (17.0)	0.832
**PPM implantation**	7 (14.6)	7 (14.6)	1.000	15 (12.6)	19 (17.4)	0.575
**Atrial fibrillation**	1 (2.1)	7 (14.6)	0.059	6 (4.8)	17 (15.3)	0.219
**New-onset LBBB**	25 (52.1)	19 (39.6)	0.219	63 (51.3)	42 (37.9)	0.218
**VT or VF**	1 (2.1)	1 (2.1)	1.000	4 (3.2)	5 (4.2)	0.776
**Pericardial effusion**	4 (8.3)	3 (6.2)	1.000	17 (14.3)	16 (14.2)	0.994
**Cardiac tamponade**	0 (0.0)	0 (0.0)	-	0 (0.0)	1 (0.9)	0.303
**BARC ≥2**	6 (12.5)	2 (4.2)	0.268	26 (21.3)	4 (3.7)	**0.007**
**Bleeding complications**			0.671			0.057
**BARC 0**	38 (79.2)	42 (87.5)		88 (71.6)	97 (87.5)	
**BARC 1**	4 (8.3)	4 (8.3)		9 (7.0)	10 (8.9)	
**BARC 2**	3 (6.2)	2 (4.2)		10 (8.4)	3 (2.7)	
**BARC 3**	2 (4.2)	0 (0.0)		14 (11.7)	1 (0.9)	
**BARC 4**	0 (0.0)	0 (0.0)		0 (0.0)	0 (0.0)	
**BARC 5**	1 (2.1)	0 (0.0)		2 (1.6)	0 (0.0)	

**Vascular complications**	2 (4.2)	3 (6.2)	1.000	16 (12.8)	6 (5.5)	0.300
**Gastrointestinal bleeding**	1 (2.1)	1 (2.1)	1.000	2 (1.6)	2 (1.8)	0.747
**Acute kidney injury**	5 (10.4)	4 (8.3)	1.000	22 (17.8)	10 (9.3)	0.321
**Renal replacement therapy**	1 (2.1)	1 (2.1)	1.000	2 (1.6)	2 (1.8)	0.686
**CPR**	1 (2.1)	1 (2.1)	1.000	5 (4.1)	3 (3.1)	0.790
**ECMO**	0 (0.0)	0 (0.0)	-	0 (0.0)	1 (0.9)	0.303
**CVA**	3 (6.2)	1 (2.1)	0.617	7 (6.1)	8 (7.4)	0.856
**Pneumonia**	2 (4.2)	2 (4.2)	1.000	6 (5.4)	9 (7.9)	0.723
**Urinary tract infection**	1 (2.1)	0 (0.0)	1.000	2 (1.6)	3 (2.7)	0.669

All values are expressed as mean ± standard deviation or the number with percentage (%).

BARC = The Bleeding Academic Research Consortium; CAVB = complete atrioventricular block; CPR = cardiopulmonary resuscitation; CVA = cerebrovascular accident; ECMO = extracorporeal membrane oxygenation; ICU = intensive care unit; LBBB = left bundle branch block; PPM = permanent pacemaker; RRT = renal replacement therapy; VF = ventricular fibrillation; VT = ventricular tachycardia.

Supplemental Table 4. Echocardiographic characteristics at discharge, 6-month, 12-month, and 24-month follow-up

	Overall (n = 134)	Elderly group (n = 61)	Super-elderly group (n = 73)	p-value
**At discharge**	**n = 130**	**n = 60**	**n = 70**	
**LVEF (%)**	64.77 ± 10.44	66.75 ± 9.61	63.10 ± 10.88	**0.046**
**Peak AoV velocity (m/s)**	2.33 ± 0.43	2.36 ± 0.46	2.31 ± 0.41	0.551
**Peak AoV PG (mmHg)**	23.99 ± 18.71	23.03 ± 9.28	24.81 ± 24.07	0.592
**Mean AoV PG (mmHg)**	11.72 ± 4.69	12.00 ± 5.13	11.48 ± 4.29	0.531
**EOA (cm^2)**	1.820 ± 0.459	1.888 ± 0.501	1.762 ± 0.414	0.119
**Indexed EOA (cm^2/m^2)**	1.156 ± 0.282	1.165 ± 0.299	1.148 ± 0.268	0.735
**Moderate to severe AR**	2 (1.5 %)	1 (1.7 %)	1 (1.4 %)	1.000
**Moderate to severe PVL**	20 (15.4 %)	7 (11.7 %)	13 (18.6 %)	0.277
**At 6-month follow-up**	**n = 101**	**n = 49**	**n = 52**	
**LVEF (%)**	65.84 ± 7.18	66.59 ± 6.91	65.13 ± 7.42	0.309
**Peak AoV velocity (m/s)**	2.27 ± 0.45	2.28 ± 0.47	2.25 ± 0.43	0.712
**Peak AoV PG (mmHg)**	21.23 ± 8.46	21.99 ± 8.95	20.51 ± 8.00	0.388
**Mean AoV PG (mmHg)**	11.24 ± 4.61	11.76 ± 5.01	10.75 ± 4.18	0.273
**EOA (cm^2)**	1.789 ± 0.407	1.798 ± 0.437	1.781 ± 0.380	0.837
**Indexed EOA (cm^2/m^2)**	1.144 ± 0.264	1.123 ± 0.277	1.162 ± 0.254	0.474
**Moderate to severe AR**	1 (1.0 %)	0 (0.0 %)	1 (1.9 %)	1.000
**Moderate to severe PVL**	10 (9.9 %)	3 (6.1 %)	7 (13.5 %)	0.320
**At 12-month follow-up**	**n = 66**	**n = 38**	**n = 28**	
**LVEF (%)**	66.22 ± 8.54	66.17 ± 9.71	66.29 ± 6.81	0.954

**Peak AoV velocity (m/s)**	2.28 ± 0.42	2.32 ± 0.46	2.23 ± 0.36	0.385
**Peak AoV PG (mmHg)**	21.54 ± 8.03	22.36 ± 8.95	20.43 ± 6.58	0.340
**Mean AoV PG (mmHg)**	11.05 ± 4.36	11.60 ± 4.96	10.31 ± 3.31	0.237
**EOA (cm^2)**	1.781 ± 0.446	1.801 ± 0.463	1.754 ± 0.429	0.671
**Indexed EOA (cm^2/m^2)**	1.135 ± 0.281	1.137 ± 0.318	1.132 ± 0.226	0.937
**Moderate to severe AR**	1 (1.5 %)	1 (2.6 %)	0 (0.0 %)	1.000
**Moderate to severe PVL**	8 (12.1 %)	4 (10.5 %)	4 (14.3 %)	0.714
**At 24-month follow-up**	**n = 37**	**n = 21**	**n = 16**	
**LVEF (%)**	65.10 ± 10.02	62.44 ± 11.93	68.76 ± 4.88	0.053
**Peak AoV velocity (m/s)**	2.30 ± 0.37	2.33 ± 0.34	2.25 ± 0.42	0.502
**Peak AoV PG (mmHg)**	21.93 ± 6.93	22.09 ± 6.72	21.71 ± 7.45	0.876
**Mean AoV PG (mmHg)**	11.69 ± 4.49	12.07 ± 4.95	11.14 ± 3.79	0.545
**EOA (cm^2)**	1.739 ± 0.374	1.690 ± 0.321	1.807 ± 0.441	0.361
**Indexed EOA (cm^2/m^2)**	1.095 ± 0.234	1.041 ± 0.191	1.171 ± 0.272	0.103
**Moderate to severe AR**	0 (0.0 %)	0 (0.0 %)	0 (0.0 %)	-
**Moderate to severe PVL**	3 (8.1 %)	0 (0.0 %)	3 (18.8 %)	0.072

All values are expressed as mean ± standard deviation or the number with percentage (%).

AoV = aortic valve; AR = aortic regurgitation; EOA = effective orifice area; LVEF = left ventricular ejection fraction; PG = pressure gradient, PVL = paravalvular leakage.
